# Case Report: Acute primary splenic torsion in a maned wolf (*Chrysocyon brachyurus)*

**DOI:** 10.3389/fvets.2025.1596698

**Published:** 2025-06-18

**Authors:** Geovanna Hernandez, Pierre P. Picavet, Aurelie Bruwier, Mariajesus Soula, Gretel Tovar-Lopez

**Affiliations:** Department of Clinical Sciences, College of Veterinary Medicine, Kansas State University, Manhattan, KS, United States

**Keywords:** splenic torsion, maned wolf, abdominal CT, blood transfusion, splenectomy

## Abstract

Splenic torsion is an uncommon condition in the Canidae family, characterized by twisting of the splenic pedicle, leading to vascular compromise. The etiology remains unclear, although one hypothesis associates it with gastric dilatation-volvulus syndrome. Splenic torsion may present acutely or chronically. Tentative diagnosis is often based on clinical signs, physical examination findings, and abdominal radiography, while ultrasonography or computed tomography confirms the condition. A 6-year-old castrated male maned wolf (*Chrysocyon brachyurus*) was presented to the Veterinary Health Center at Kansas State University with an acute onset of anorexia, lethargy, and apparent ataxia. Physical examination revealed pale, moist mucous membranes, a firm and large mass occupying most of the cranial abdomen, and suspected abdominal effusion. Abdominal ultrasound and computed tomography confirmed an acute splenic torsion. Emergency splenectomy was performed, and diagnosis was confirmed grossly and subsequently by histopathologic study. The patient received a xenotransfusion with one unit of canine packed red blood cells and two units of canine plasma intraoperatively. Recovery was uneventful, and no complications were noted 7 months postoperatively. This case report documents the first recorded instance of acute primary splenic torsion in a maned wolf. Diagnostic imaging findings were comparable to those seen in domestic dogs, and similar therapeutic approach resulted in a successful outcome.

## Introduction

Splenic torsion (ST) is an uncommon pathology in the Canidae family that involves twisting of the splenic pedicle, resulting in vascular obstruction. Venous outflow is occluded first, causing congestion, followed by arterial compromise, leading to infarction (1, 2). The etiology of ST remains uncertain, but in dogs, it is hypothesized to be associated with or result from gastric dilatation-volvulus (GDV), which may stretch or loosen the gastrosplenic ligaments, allowing abnormal splenic mobility. It may also leave the spleen torsed after spontaneous resolution. Alternatively, ST itself may predispose patients to GDV by disrupting normal gastric motility (1, 3).

Clinical signs of acute ST in dogs often reflect hypovolemic or endotoxic shock due to compromised perfusion and systemic inflammation (1). Chronic cases tend to present more subtly, with nonspecific clinical signs such as lethargy, anorexia, vomiting, diarrhea, polyuria, polydipsia and weight loss (1, 2, 4). A presumptive diagnosis may be made based on clinical signs, physical examination findings, and radiographs, but preoperative definitive diagnosis typically requires abdominal ultrasonography (US) with Doppler or computed tomography (CT) (2, 5, 6).

Abdominal ultrasound and CT findings in dogs with ST often share similar characteristics. Ultrasonographic findings in dogs with ST include generalized splenomegaly, splenic congestion, abnormal splenic position, parenchyma hypoechogenicity, absent or reduced blood flow, hyperechoic perisplenic fat with hilar perivenous triangles, and hemorrhagic peritoneal effusion (6, 7). CT findings include an enlarged spleen with rounded margins, and a folded C shape, absence of contrast enhancement, a “whirl sign” of torsed vessels and gastro/phrenosplenic ligaments, associated with a hyperattenuating center on pre-contrast images and the presence of free abdominal fluid (6).

Splenectomy is the treatment of choice and preoperative stabilization is critical, particularly in cases with hypovolemic shock secondary to hemoabdomen. Manipulating or untwisting the spleen before removal is discouraged, as this may release thrombi, sequestered blood, or inflammatory mediators into circulation (1).

Prognosis in dogs ranges from guarded to good and is generally more favorable in chronic cases due to lower risk of shock and toxemia (2, 4). Prophylactic gastropexy is recommended in dogs undergoing splenectomy due to the increased risk of GDV (2, 8), though the incidence of GDV in maned wolves remains undocumented (9). One case series of six maned wolves diagnosed with GDV reported that three of them had concurrent ST, suggesting a secondary cause (9). However, no prior reports describe primary splenic torsion in this species. This case represents the first such instance, managed successfully using diagnostic and therapeutic approaches adapted from domestic canine medicine.

## Case report

A 6-year-old neutered male maned wolf (*Chrysocyon brachyurus*), housed at a zoological facility in Manhattan, Kansas, was presented to the Kansas State University Veterinary Health Center following a 12-h history of anorexia, apparent ataxia, lethargy, and unresponsiveness to mild physical stimuli, as observed by the caretaker.

At the time of the patient's annual examination, a complete blood count (CBC), serum chemistry, urinalysis, and serologic testing for *Anaplasma, Borrelia*, and *Ehrlichia* antibodies, as well as heartworm antigen testing via ELISA (SNAP 4DX, IDEXX), were performed. The CBC revealed erythrocytosis [hematocrit (Hct) 59%; reference range (RR) 29–53.9%], while serum chemistry and infectious disease screening were unremarkable. Urinalysis showed 3+ bilirubin, 3+ protein, and increased cystine crystals.

Due to acute clinical deterioration, the patient was isolated and immobilized via intramuscular (IM) blow dart administration of ketamine 6 mg/kg, midazolam 0.3 mg/kg, and butorphanol 0.5 mg/kg. After 15 min, the patient remained responsive to stimuli so, an additional ketamine dose (2 mg/kg IM) was given by hand injection. The patient was intubated with a 15 mm endotracheal tube and maintained on 1.5% isoflurane with 100% oxygen.

On physical examination, mucous membranes were pale and moist. A large mass occupying most of the cranial abdomen and asuc palpable caudal abdominal effusion were noted. All other parameters were within normal limits. A CBC revealed mild anemia (Hct 21%; 29–53.9%), severe thrombocytopenia (36 K/μL; 106–690 K/μL), neutrophilia (18.38 K/μL; 0.01–11.95 K/μL) and monocytosis (2.7 K/μL; 0–1.1 K/μL). Serum biochemistry results were unremarkable.

**Figure 1:**
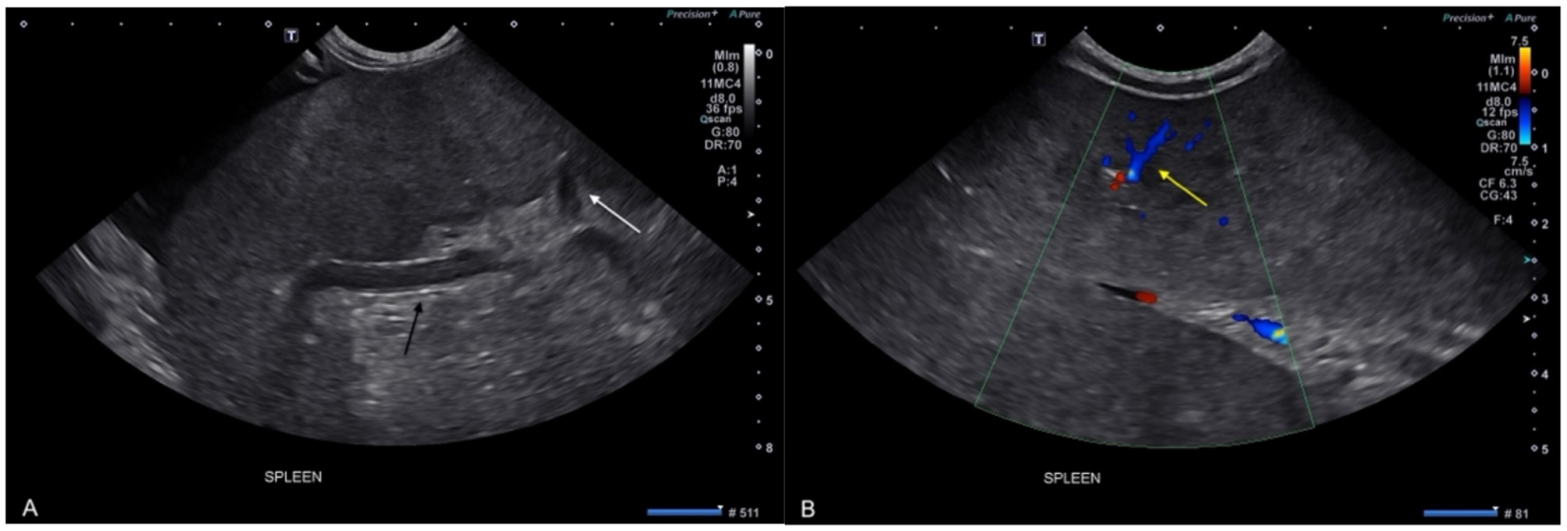
Ultrasound images of a maned wolf (*Chrysocyon brachyurus*) with primary splenic torsion **(A, B)** showing an enlarged spleen with a moderately heterogeneous parenchyma, hyperechoic adjacent fat and hyperechoic perihilar triangular regions (white arrow), as well as splenic vein thrombosis (black arrow) and peritoneal effusion **(A)**. On color Doppler examination **(B)**, some blood flow was still visible (yellow arrow).

Abdominal US revealed a moderate amount of slightly particulate anechoic peritoneal effusion. The spleen was markedly enlarged, folded on itself, with rounded margins and diffusely heterogenous parenchyma containing mildly hypoechoic areas. The surrounding fat was hyperechoic with hilar perivenous hyperechoic triangles. Echogenic material was noted in the splenic veins, and Doppler examination showed absent or markedly reduced venous flow (Figure 1). Findings were suggestive of ST with splenic vein thrombosis. Mild to moderate functional ileus was also observed. Crystalluria and small urinary bladder stones were present. These were considered incidental findings as were also noticed unchanged from previous healthy exam, and there were no clinical signs of urinary obstruction or secondary infection, and addressing the splenic torsion was prioritized over elective cystotomy in this emergency setting.

**Figure 2:**
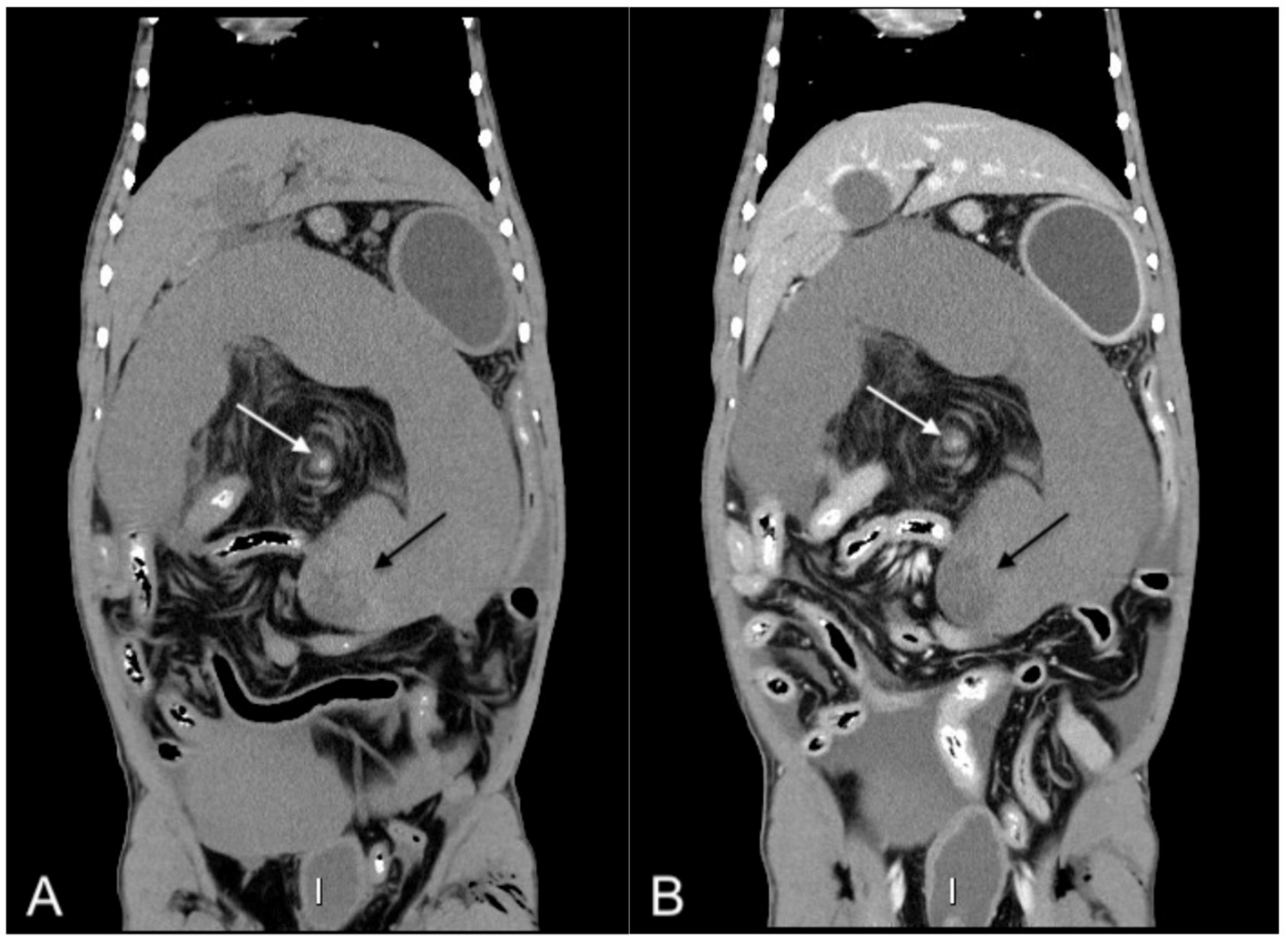
Dorsal plane pre- **(A)** and postcontrast **(B)** computed tomographic images of the abdomen, displayed in soft tissue window, showing the enlarged folded C-shaped spleen, the lack of contrast enhancement of the splenic parenchyma, the “whirl sign” (white arrow) associated with a hyperattenuating center visible on pre-contrast images. A region of splenic infarction is pointed by the black arrow.

Due to the uncommon nature of ST, an abdominal CT scan was performed to confirm the diagnosis and rule out any concurrent pathology. CT revealed a torsed, markedly enlarged spleen with rounded margins and lack of parenchyma or vascular contrast enhancement [45 Hounsfield units (HU) pre-constrast and 46 HU post-contrast]. A “whirl sign” was noted at the level of the splenic vasculature with a hyperattenuating center on pre-contrast images (Figure 2). A hypoattenuating, non-enhancing region within the splenic head (25 HU) and peritoneal effusion (26 HU, suggesting hemorrhagic fluid) were observed. Urinary bladder findings like those described with US were also noted. Gastric position was normal, and no evidence of concurrent disease was found. CT findings confirmed splenic torsion with splenic infarct, thrombosis, and hemoabdomen.

The patient underwent emergency splenectomy. The patient was placed in dorsal recumbency and midline celiotomy was performed. The spleen was identified as torsed, congested, and dark with multifocal parenchymal fractures but no active hemorrhage. The left limb of the pancreas was adjacent to the torsed splenic pedicle. A Carmalt forceps was placed proximal to the torsion and distal to the pancreas. The pancreas was carefully dissected free from the omental leaf. Three ligations using 0 polydioxanone suture (PDS, Ethicon) were placed on the splenic pedicle, avoiding pancreatic vessels. Cautery was applied to all vessel tips. A prophylactic incisional gastropexy was performed with 2-0 barbed Glycolide-dioxanone-trimethylene carbonate suture **(**V-Lok CoViden suture). The abdomen was lavage with warm saline and the *linea alba* was closed using 1-0 polydioxanone suture (PDS, Ethicon) in a simple continuous pattern. The subcutaneous layer was sutured with 2-0 polydioxanone suture (PDS, Ethicon) in a simple continuous pattern, while the dermis was closed with 2-0 polyglecaprone suture (Monocryl, Ethicon) in the same manner. Skin glue was applied over the incision site to secure the closure.

A xenotransfusion with one unit of DEA- negative canine packed red cells at 21 ml/kg/h followed by two units of canine plasma were administered at the same rate during surgery due to anemia and hypotension. A norepinephrine constant rate infusion (0.1 μg/kg/h) was also administered. No adverse reactions to the xenotransfusion or plasma were observed. A single dose of ampicillin and sulbactam (30 mg/kg IV) was given.

The patient was maintained hospitalized for 24 h. Postoperative management included intravenous (IV) fluids (Lactate Ringer solution at 3 ml/kg/h), hydromorphone (0.05 mg/kg IV bolus followed by a constant rate infusion at 0.02 mg/kg/h), maropitant citrate (1 mg/kg IV once), pantoprazole (0.7 mg/kg IV every 12 h), meloxicam (0.01 mg/kg subcutaneously every 24 h), and ondansetron (0.5 mg/kg IV every 12 h).

The following day, the patient was stable and pain-free. A CBC showed a Hct of 25% and persistent neutrophilia (20 K/μL; 0.01–11.95 K/μL). Biochemistry showed a marked elevation of creatinine kinase (11,304 μL; RI: 50–470 μL), while other values were unremarkable. The patient was discharged and returned to the zoological facility for close observation.

Gross examination of the spleen revealed firm, tan intraparenchymal areas and a raised capsular lesion on the diaphragmatic surface. Histopathology confirmed splenic torsion with fibrin deposition, hemorrhage, and mild mineralization. No evidence of infectious agents or neoplasia were detected.

Forty-eight hours postoperatively, the wolf developed diarrhea and anorexia. Meloxicam was discontinued. Pain indicators including quiet mentation, growling, hunched posture, and reluctance to move, were observed. Buprenorphine (3 mg/kg transdermal as a single dose) and maropitant citrate (1 mg/kg IM as a single dose) were given. Sucralfate (37 mg/kg orally every 8 h) was prescribed. Clinical signs resolved within 24 h. Sixteen days post-op, the patient was sedated using the same protocol previously described for re-evaluation. Physical exam, CBC and serum biochemistry were normal, and the surgical site had healed.

Seven months post-surgery, the maned wolf remained clinically normal with no reported complications.

## Discussion

This case report describes the diagnosis and successful management of acute primary splenic torsion in a maned wolf (*Chrysocyon brachyurus*), a condition not previously reported in this species. The etiology of splenic torsion in canine family remains uncertain, with potential causes including inflammatory disease processes, congenital splenic ligament laxity (commonly referred to as a “wandering spleen”), acquired laxity secondary to spontaneously resolved GDV, neoplasia, peritonitis, accessory splenic tissue, and trauma (1, 4, 10). The absence of concurrent GDV along with the absence of gastric abnormalities typically associated with resolved GDV during exploratory laparotomy (e.g., overdistension, congestion, or signs of tissue ischemia or necrosis), neoplasia, or trauma suggests a truly primary etiology. This distinguishes the case from prior reports, where splenic torsion occurred secondarily to GDV in maned wolves (9). Although splenomegaly can be caused by several infectious and inflammatory diseases in wild canids, the clinical and histopathologic findings in this case were consistent with an acute vascular event and not indicative of systemic infectious disease.

Infectious canine hepatitis caused by canine adenovirus type 1 (CAV-1) has been reported in maned wolf litters and is known to cause splenomegaly and hepatomegaly (11). However, this patient showed no clinical signs or histopathologic changes indicative of viral hepatitis. The wolf remained normothermic and exhibited no evidence of hepatic dysfunction on bloodwork or imaging. Additionally, as an adult not recently part of a litter, the likelihood of an acute systemic viral infection was considered low, since such infections are primarily observed in juvenile individuals.

In exotic species in captivity, early identification of disease is often delayed due to their tendency to mask clinical signs until the condition becomes advanced. In this case, the acute presentation of anorexia, lethargy, followed by prompt diagnostic imaging, enabled timely surgical intervention. The diagnosis of acute primary splenic torsion (ST) in this case was supported by several key findings, including the sudden onset of clinical signs and the absence of predisposing factors such as gastric displacement or adhesions observed during exploratory surgery (10). In contrast, a case report describing chronic ST in a dog noted intraoperative findings of adhesions involving the duodenum and omentum, with fibrous tissue containing vessels up to 1.0 mm in diameter. Some of these adhesions were fibrinous, particularly around the omentum, supporting the diagnosis of a chronic process (4).

Abdominal CT is a valuable diagnostic exam for patients presenting with clinical signs suggestive of ST. Although CT might be considered more invasive than abdominal US, it is often quicker to perform and provides a more extensive evaluation. CT scan can facilitate a thorough examination of the abdominal cavity, helping to exclude other potential pathologies that might be challenging to diagnose with ultrasound on a large patient. CT typically requires the patient to be under anesthesia to ensure optimal imagining quality. However, this may not always be feasible or advisable, particularly in critical patients. In cases involving exotic animals, as illustrated in this instance, anesthesia is generally essential, as most diagnostic procedures cannot be conducted safely without it due to patient behavior and the need to ensure staff safety (12).

Ultrasonographic and CT findings in this case closely resembled those reported in domestic dogs. Ultrasound revealed decreased or absent Doppler flow, heterogenous parenchyma, and peri splenic hyperechoic fat-hallmark features of ST (5, 7). As congestion, hemorrhage, or infarction progresses, the splenic parenchymal echogenicity and echostructure may vary (13). CT demonstrated the classic “whirl sign” of twisted splenic vasculature, minimal contrast enhancement, and a folded C- shaped spleen. A median difference between pre- and postcontrast parenchymal attenuation of +1HU was noted, like previously published findings (+1.15 HU), consistent with a lack of splenic parenchymal enhancement (5). In our case, an area of splenic infarction was also visible. The absence of gastric displacement or ischemic signs further supported a diagnosis of primary splenic torsion. These similarities support the extrapolation of diagnostic imaging criteria from dogs to maned wolves.

The presented maned wolf developed moderate anemia due to hemoabdomen and intraoperative hypotension, likely secondary to blood loss and the vasodilatory effects of inhalant anesthesia and opioids, as commonly observed in similar cases involving abdominal hemorrhage (14). Blood transfusion likely benefited the patient by minimizing post-surgical complications and improving the overall prognosis. In dogs with splenic torsion and hemoabdomen, it is crucial to have blood units readily available. However, such resources may not always be accessible in basic care units, such as zoos or small hospitals, emphasizing the need for improved preparedness in managing critical cases. In this case, a xenotrasfusion using dog blood (DEA -) was used. A study conducted to detect the presence of canine erythrocyte antigen 1 (DEA 1) and to evaluate compatibility between domestic dogs and non-domestic canids, including the maned wolf, revealed that all tested individuals in the study were negative for DEA 1. The absence of DEA 1 in maned wolves indicates a reduced risk of adverse reactions during blood transfusion, as this antigen is a leading cause of hemolytic transfusion reactions in DEA1 positive domestic dogs (15). No further information was found regarding xenotransfusions in this species.

A previous study looking for the association between splenectomy and the subsequent development of GDV in dogs reported a high incidence risk of GDV in patients that underwent splenectomy (10, 16). Prophylactic gastropexy is thus recommended, particularly in cases involving splenic torsion or splenectomy, especially in patients predisposed to GDV for example, large or giant breed or deep chested dogs (16). The maned wolf, as indicated in a case report documenting six cases of GDV in captive individuals, is considered a species at increased risk due to its anatomical conformation (9). Consequently, prophylactic gastropexy was performed in the presented patient. To date, 7 months post-surgery, no clinical signs of GDV have been observed.

The patient exhibited common post-operative complications observed in dogs undergoing exploratory laparotomy, including diarrhea, likely associated with medication administration and/or stress. However, abdominal ultrasound and CT imaging did not reveal any abnormalities suggestive of gastrointestinal tract involvement. This complication resolved within 24 h, and no additional treatment was necessary.

## Conclusion

This case documents the first known instance of acute primary splenic torsion in a maned wolf. The absence of predisposing conditions such as GDV, trauma, or neoplasia, along with diagnostic imaging and histopathologic findings, supports this classification. While other causes of splenomegaly in this species, such as infectious canine hepatitis, have been reported, this case did not exhibit the systemic symptoms or tissue abnormalities commonly seen in those diseases. A rapid diagnostic approach and timely surgical intervention contributed to a positive outcome. This case highlights the utility of diagnostic imaging adapted from domestic dogs, the potential life-saving role of xenotransfusion, and the need for continued vigilance for rare but critical conditions in nondomestic canids.

## Data Availability

The raw data supporting the conclusions of this article will be made available by the authors, without undue reservation.
